# Supplementary material for: Src-Dependent NM2A Tyrosine Phosphorylation Regulates Actomyosin Remodeling
Source: Cells. 2023 Jul 17;12(14):1871. doi: 10.3390/cells12141871 (PMC10377941; doi:10.3390/cells12141871)
Supplement: Supplementary file 1 [file cells-12-01871-s001.zip › cells-2467142-supplementary.pdf]

Supplementary Materials: "Src-dependent NM2A tyrosine-phosphorylation regulates actomyosin dynamics"

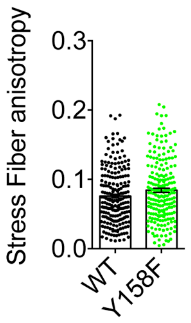

Figure S1. Quantification of the anisotropic distribution of stress fibers in HeLa cells ectopically expressing GFP-NMHC2A-WT or Y158F. Each dot corresponds to a single cell. Values are the mean ± SEM (n > 200)

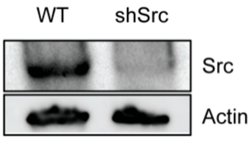

Figure S2. Immunoblots showing Src and actin levels in extracts from WT and shSrc HeLa cells. Actin was used as loading control.

Table S1. shRNA sequences for Src knockdown in HeLa cells.

**shRNA Sequences (5′-3′)**		**Source**
shControl-SHC016	CCGGGCGCGATAGCGCTAATAATTTCTCGAGAAA TTATTAGCGCTATCGCGCTTTTT	Sigma
shSrc-TRCN0000023597	CCGGGTGGCTTACTACTCCAAACATCTCGAGATGT TTGGAGTAGTAAGCCACTTTTT	

Movie S1. Ectopic expression of the different GFP-NMHC2A variants affects cell motility in HeLa cells. HeLa cells ectopically expressing GFP-NMHC2A-WT or -Y158F were followed by time-lapse microscopy for more than 15 h. (Upper panel) Sequential frames acquired every 10 min (8 frames per second display rate). Overlay of the transmitted light and the fluorescence of GFP-NMHC2A variants is shown. Scale bar, 50 μm. (Lower panel) Representative tracking of the movement of cells expressing WT or Y158 versions of NMHC2A, produced by the Manual Tracking plug-in on Fiji™. Scale bar, 50 μm.
